# Prediction of clinical outcomes in individuals with chronic low back pain: a protocol for a systematic review with meta-analysis

**DOI:** 10.1186/s13643-018-0818-2

**Published:** 2018-10-02

**Authors:** Liliane Mendonça, Matilde Monteiro-Soares, Luís Filipe Azevedo

**Affiliations:** 1 OBSERVDOR - Centro Nacional de Observação em Dor / NOPain - National Observatory for Pain, Porto, Portugal; 2 CINTESIS - Centro de Investigação em Tecnologias e Serviços de Saúde, Rua Dr. Plácido da Costa, s/n, 4200-450 Porto, Portugal; 3 MEDCIDS - Departamento Medicina da Comunidade, Informação e Decisão em Saúde, Oporto, Portugal

**Keywords:** Systematic review, Chronic low back pain, Prognostic, Multivariate models

## Abstract

**Background:**

Low back pain (LBP) is one of the most prevalent and recurrent conditions in the general population, with personal, professional, social and economic impact. However, there is a lack of consistent evidence about chronic low back pain (CLBP) prognosis, especially highlighting predictors that influence CLBP outcome. Existing systematic reviews are scarce, outdated and incomplete. The primary aim of this systematic review is to identify multivariable models and/or predictors associated with clinical outcomes in subjects with CLBP (namely pain intensity, disability, return to work, psychological well-being and quality of life).

**Methods:**

We will systematically search Ovid MEDLINE (PubMed), Scopus and Web of Science databases for longitudinal studies, published until June 2017, including adults with CLBP (defined as persistent pain with ≥ 3 months duration), which studied the association between multivariable models and/or predictors with at least one of the selected clinical outcomes after ≥ 3 months of follow-up. Articles’ screening and selection will be conducted by two reviewers, blindly and independently. Disagreements will be resolved by a third reviewer. Models’ discriminative ability will be assessed using *C*-statistic. The link between multivariable models and predictors with the clinical outcome will be analysed through association measures. Qualitative and quantitative synthesis of the available evidence will be performed. Meta-analysis will be conducted to aggregate each type of measure. In the absence or in the presence of only slight to moderate of heterogeneity, we will use the fixed or random effects model, respectively. In case of moderate to severe heterogeneity, an attempt to explain variability in detail will be made through subgroups and sensitivity analyses. Subgroup analysis will be conducted according to clinical outcome, follow-up duration (≤ 6 months versus > 6 months) and type of context (pain management clinics versus other therapeutic settings).

**Discussion:**

We consider that it is urgent to highlight the available evidence about CLBP prognosis. This systematic review will help identify multivariable models and individual predictors that may enhance pain management success. One potential limitation will be the difficulty of aggregating quantitative measures from several prognostic models and predictors, with different clinical outcomes.

**Systematic review registration:**

PROSPERO CRD42017079233

**Electronic supplementary material:**

The online version of this article (10.1186/s13643-018-0818-2) contains supplementary material, which is available to authorized users.

## Background

Low back pain (LBP) is one of the most prevalent types of chronic pain in clinical practice [1]. Researchers estimate that low back pain affects 4–33% of the population at any given point, and will affect 60–80% of the population at some point during life, and this prevalence increases with age [2–4]. After an acute low back pain episode, the majority (about 90%) recovers in a few months [5, 6], although recurrences are common (varying between 25 to 50% in a year) [7]. Furthermore, LBP recurrences are the main occurrence responsible for seeking health care, sick leave and other work-related problems and activity limitations, which lead to a major impact on financial and human resources [7]. Definitions of onset or conclusion of an acute or subacute low back pain episode and what is considered recovery are still unclear [8].

When low back pain persists for more than 3 months, it is considered to be a chronic low back pain (CLBP) [9, 10]. Although the progression to CLBP occurs in a minority of the population with low back pain (about 10% of the adult population [10]), CLBP is a complex and heterogeneous condition, disabling and costly [11], with an uncertain prognosis [8, 12]. So, objective measures have been studied to accurately predict low back pain progression from acute to chronic. The most commonly used variables are work-related (workers’ litigation status, return to work), clinical (treatment interventions), psychological (psychiatric comorbidities, health-related quality of life, and coping behaviors), and sociodemographic [13–15].

Until now, much more focus has been given to acute or subacute low back pain episodes’ prognosis when compared to CLBP. However, acute and chronic low back pain natural courses differ greatly, and it is possible that the prognostic factors are also different [16]. For instance, individuals with CLBP are more likely to seek and use health care resources than individuals with acute low back pain episodes [5]. Thus, we consider that more attention should be given also to CLBP prognosis.

Highlighting predictors that influence the clinical outcome of CLBP is a major challenge to improve prognosis and select patients for which specific therapies may be required, still, there is a lack of consistent evidence around CLBP prognosis. The most effective way of translating evidence into clinical care is through clinical prediction rules, meaning, multivariate models that stratify subjects by their risk of having or developing a determined clinical outcome, or by their chance of benefit from a specific treatment [17]. However, one of the crucial steps for these models’ derivation and assessment is to understand which composing variables are most pertinent [18].

To the best of our knowledge, there are few systematic reviews [16, 19–21] focusing on prognostic multivariable models and/or individual predictors for CLBP that are clearly outdated and have provided a less comprehensive and broad perspective around this topic. The most recent review included studies published until 2010 [16] and the remaining included studies from more than 15 years back [19–21]. Another concern is that they had study type and/or participants’ restrictions which can impair the results’ generalization, for example, including only randomized controlled trials [16] or subjects undergoing multidisciplinary rehabilitation [20].

For all this, with our review, we primarily aim to identify multivariable models used to predict clinical outcomes in subjects with CLBP, namely pain intensity, disability, return to work, psychological well-being and quality of life (QoL).

As we expect to find few published multivariable models for these outcomes’ prediction, in order to guide future research and models’ development, we also aim to ascertain isolated univariate predictors that are linked to CLBP patients’ prognosis, meaning the probability of improving clinical outcomes during follow-up. So, we will focus on independent univariate predictors’ and multivariable models association with the described clinical outcomes, meaning we aim at identifying studies both with univariate and/or multivariable analysis.

Depending on several study characteristics, such as duration of follow-up after LBP chronicity has installed, clinical setting and methodological quality, the results achieved may be highly heterogeneous. To better understand the impact of such different characteristics between studies, we aim to explore them in detail. In addition, we aim to enumerate the instruments most commonly used to assess CLBP prognosis and measure pain intensity, disability, return to work, psychological well-being and QoL in individuals with CLBP.

## Methods/ Design

This review protocol was registered in PROSPERO database under the number CRD42017079233.

This study protocol was defined following the recommendations and orientations included in the PRISMA-P (Preferred Reporting Items for Systematic review and Meta-Analysis Protocols) checklist [22].

### Search strategy

The search will be carried out in Ovid MEDLINE (PubMed), Scopus and Web of Science databases. The construction of the query search will be an iterative process in which controlled vocabulary, free text, synonyms and related terms will be used, connected by Boolean operators. The used search is described in Additional file 1: Table S1. We have used a broad query in order to achieve the highest sensitivity possible. Thus, we have not restricted the search by date limit, context, any specific chronic low back pain etiology (such as neuropathic, oncological, etc.), follow-up, study type or language.

Our search will be complemented by manual searching abstracts books of relevant congresses and scientific meetings held in the last 10 years [namely IASP (International Association for the Study of Pain), EFIC (European Pain Federation) and World Institute of Pain], in addition to the references of included studies and other related reviews.

### Inclusion and exclusion criteria

Articles will be included if they were:

- Published until June 2017
- Clinical trials or observational studies (such as prospective, retrospective, cohort or case-control studies),
- Conducted on adults (> 18 years) with chronic low back pain (defined as persistent pain that lasts for more than 3 months),
- Evaluated the predictive ability of multivariable models or individual predictors and the strength of association between outcomes and predictors with pain intensity, or disability, or return to work, or psychological well-being or QoL and
- Had at least 3 months of follow-up.

Articles will be excluded if they:

- Included a population with a specific pathology (namely arthritis, fractures, nerve compression) or
- Had as a primary goal to assess the efficacy of a specific intervention (whether pharmacologic or another kind).

No restriction on the language of publication will be applied.

### Procedure

The screening procedure will firstly focus on titles and abstracts of the articles found. Secondly, all potential studies will be selected through full-text reading. Both procedures will be carried out by two reviewers (LM and MMS), blindly and independently.

All reasons for exclusion will be recorded. Disagreements will be resolved by a third reviewer (LFA). Reliability of the selection process will be evaluated using a proportion of agreement and kappa statistics.

Authors of the selected studies will be contacted whenever additional clarifications are necessary regarding inclusion criteria fulfillment or data extraction.

We will describe the procedure of identification, study selection and data analysis using a PRISMA flow chart identical to the one presented in Additional file 1: Figure S1.

### Assessment of study bias

Methodological quality and risk of bias assessments will be performed by two reviewers (LM and MMS), blindly and independently. Disagreements will be resolved by a third reviewer (LFA). The agreement proportion and kappa statistic will be used to evaluate the reliability of the process. We will use the CHARMS (CHecklist for critical Appraisal and data extraction for systematic Reviews of prediction Modelling Studies) [23] and TRIPOD (Transparent reporting of a multivariable prediction model for individual prognosis or diagnosis: The TRIPOD statement) [24] checklists to assess study quality and risk of bias. This checklist is the most frequent and appropriate to use for systematic reviews of multivariable and predictors of clinical outcomes. It was created by the Cochrane Prognosis Methods Group and mainly focus on the source of data; participants’ selection and characterization; outcomes’ and predictors’ definition and measurement; sample size; missing data; description of methods to develop, assess the performance and/or evaluate the predictive model per se; results description and interpretation. Each item of the checklist will be scored as 1 in the case of full fulfillment and of 0 in the absence of the required information. The sum of the score will be used to assess the study global methodological quality, as it represents the number of items present.

### Data extraction and synthesis

Data will be collected through a specific form (Additional file 1: Tables S2 and S3) including the general characteristics of the study (such as study design, sample size, follow-up duration and measurement points), sample socio-demographic characteristics (namely age, gender, context, chronic pain definition), predictors or multivariable models analysed, discriminative ability and association measures, outcomes’ measurement methods and results, lost to follow-up and missing data.

For both independent predictors and multivariable models, association measures will be collected (namely relative risk, odds ratio, hazard ratio) in addition to their respective 95% confidence intervals or standard error. We will also collect their discriminative ability measures, namely *C*-statistic, and their respective 95% confidence intervals.

Whenever possible, the raw data will be extracted, allowing the calculation of the respective effect measure (if necessary, studies’ authors will be contacted). If it is not possible to collect these data, a measure of effect and its precision will be extracted.

In all studies, the number of participants and events will also be recorded.

Like the selection phase, the extraction will be carried out by two reviewers (LM and MMS), blindly and independently, and disagreements will be resolved by a third reviewer (LFA).

Covidence systematic review software (Veritas Health Innovation, Melbourne, Australia. Available at www.covidence.org) will be used in the screening phase and data extraction. We have also used this software to detect possible duplicates. The removal of such duplicates will be made individually and carefully, assessing if it was adequately identified.

As this review will analyse the value of multivariable models or predictors for predicting pain management outcomes, articles will be ordered by study type and CHARMS and TRIPOD scores.

### Analysis

A descriptive analysis of studies characteristics, methodologic quality, vote counting of studied multivariable models or individual predictors and statistically significant associations will be performed.

Meta-analysis will be attempted in the case we find two or more articles reporting the association between a specific or a group of variables and one of the CLBP clinical outcomes. With the help of Cochrane collaboration RevMan v.5.1® and Meta-DiSc software, data extracted from the primary studies will be analysed to perform quantitative synthesis through meta-analysis in order to obtain aggregate multivariable models’ and individual predictors’ association with clinical outcomes and also discriminative ability measures in the case of prognostic models.

We will calculate and present pooled odds ratio for predictors and *C*-statistic for multivariable models. To do so, we will use a random effects model. We have selected this model as we expect to have studies with widely different methodologies, designs and outcomes. Thus, we will conduct separate meta-analyses by study type and clinical outcome.

Heterogeneity will be evaluated using the Cochran *Q* test (significance level of 0.05), supplemented by the *I*^2^ statistic. We will consider the absence of heterogeneity as an *I*^2^ < 5% and/or a non-significant Cochran *Q* test, and slight to moderate heterogeneity as 5% < *I*^2^ < 50%).

In case of severe heterogeneity (*I*^2^ > 50%) [25], we will proceed to subgroup analysis by type of clinical outcome (pain intensity, disability, return to work, psychological well-being and QoL), follow-up duration (short versus long duration, defined as equal or inferior and superior to 6 months respectively [16], type of context (pain management clinics versus other therapeutic settings) and population characteristics (such as age). Also, meta-regression will be conducted, if there are at least five primary studies included in the analysis. Meta-regression will also be used to explore heterogeneity when there are at least five primary studies included.

The existence of publication bias will be evaluated through the construction of funnel plots.

## Discussion

CLBP is a complex condition caused and/or worsened by a myriad of factors [16] that create a heterogeneous population for which the same treatment will present different results [26]. To understand how these factors affect CLBP prognosis is of paramount importance to counsel patients, plan and select therapeutic programmes as well as assess these programmes’ efficacy.

There are several systematic reviews and meta-analyses focusing on acute and subacute LBP prognosis and evolution to chronicity. On the other hand, to our knowledge, there are only five systematic reviews regarding CLBP prognosis [16, 19–21, 26, 27] that present important limitations. The first systematic review being published on this topic focused only on predicting the return to work [20]. More recent systematic reviews focused on predictors’ impact again only in just one specific clinical outcome in CLBP, such as disability and return to work, respectively [19, 21]. One of the most comprehensive and broad reviews included studies only until 2003 [20]. In the most recently published, the authors have only included randomized controlled trials published until 2010 [16]. Multivariable prognostic models were not reported in any of these systematic reviews. For all this, we considered that an up-to-date and broader view of the available evidence about this topic is urgent. Having this in mind, we have conducted this systematic review mostly trying to avoid criteria that would constrict available evidence identification and inclusion.

In an attempt to have the most global vision of all the evidence that exists around this topic and be able to discuss it and understand which should be the next steps for adequate future research, we chose not to select the population, study type (by design or methodological quality), etc. However, in order to put into perspective the studies and results presented, we will include a complete assessment of the study quality and risk of bias for all the included studies.

With our systematic review, we will address for the first time prognostic multivariable models’ and composing univariate predictors’ pertinence, include new evidence produced in the last 7 years and focus on subjects undergoing generic interventions or multidisciplinary programmes that better represent the clinical course of this condition, this is, without the effect of specific therapies.

We have excluded studies assessing the efficacy of specific interventions from our systematic review because, in the first place, we are interested in the prognosis of CLBP patients in real-life settings where such a prediction is more meaningful and, in general, informative. Efficacy studies are mostly experimental, comparative, interventional and controlled randomized trials, typically focusing on the assessment of short-term outcomes and linked to a given intervention. The conditions in which these studies are undertaken, in general, are very different from those of real-life, usual care, and patient’s experiences. So, they will not resemble or be adequately a representative of the prognosis of real-life CLBP patients. In the second place, we are primarily interested in predicting the prognosis of CLBP patients and not specifically in the efficacy of any given intervention. Efficacy studies generally present data very much focused on the assessment of the efficacy of the intervention and not necessarily representative of the “natural” prognosis of CLBP patients’ population. In addition, it would be impossible to include all the interventions assessed in this context.

Our main goal was to answer to one of the recommendations of the Report of the National Institutes of Health Task Force that states that studies should “improve prognostic stratification of patients with CLBP” [9]. Therefore, this systematic review will identify and summarize the multivariable models and predictors of CLBP, enhancing pain management success by presenting, summarizing and discussing the best prognostic stratification tools in this context. Thus, this systematic review is expected to have, direct or indirect, major clinical impact.

A potential limitation of this study will be the difficulty of aggregating quantitative measures from several multivariable models and predictors since the different studies measure a wide range of clinical outcomes with a large diversity of instruments. But, on the other hand, it will allow the presentation and discussion of a comprehensive overview of the complexity of CLBP condition and its prognosis.

## Additional file

Additional file 1: **Table S1.** Search terms used in the different search engines. **Table S2.** Extraction form for predictors. **Table S3.** Extraction form for multivariable models. **Figure S1.** Future PRISMA flow chart for the systematic review process. (DOCX 46 kb)

## Abbreviations

CHARMS: CHecklist for critical Appraisal and data extraction for systematic Reviews of prediction Modelling Studies
CLBP: Chronic low back pain
CP: Chronic pain
EFIC: European Pain Federation
IASP: International Association for the Study of Pain
LBP: Low back pain
PRISMA-P: Preferred Reporting Items for Systematic review and Meta-Analysis Protocols
QoL: Quality of life
TRIPOD: Transparent reporting of a multivariable prediction model for individual prognosis or diagnosis: The TRIPOD statement
