# Supplementary material for: Prediction of clinical outcomes in individuals with chronic low back pain: a protocol for a systematic review with meta-analysis
Source: Syst Rev. 2018 Oct 2;7:149. doi: 10.1186/s13643-018-0818-2 (PMC6169105; doi:10.1186/s13643-018-0818-2)

# Additional file 1

Table S1 Search terms used in the different search engines

| **PICO structure** | **Ovid MEDLINE (Pubmed)** | **Scopus** | **Web of Science** |
| --- | --- | --- | --- |
| Population | #1 (("Chronic Pain"[Mesh]) OR (Chronic OR Persist*) AND Pain[ti]) | #1 TITLE-ABS-KEY (pain) AND TITLE-ABS-KEY (chronic OR persist*) | #1 (pain AND (chronic OR persist*)) |
| Intervention/ Comparator | #2 (prognos*[Text Word] OR predict*[Text Word]) | #2 TITLE-ABS-KEY (prognos* OR predict* ) | #2 Topic: (prognos* OR predict*) |
| Outcomes | #3 ("Quality of Life"[Mesh] OR pain redu* [Text Word] OR “Exercise” [Mesh] OR wellbeing OR emotional OR interference OR severity OR intensity OR disability) | #3 TITLE-ABS-KEY (quality AND of AND life OR pain AND redu* OR exercise OR wellbeing OR emotional OR interference OR severity OR intensity OR disability) | #3 Topic: (Quality of Life OR pain redu* OR Exercise OR wellbeing OR emotional OR interference OR severity OR intensity OR disability) |
|  | #1 AND #2 AND #3 | #1 AND #2 AND #3 | #1 AND #2 AND #3 |

Table S2 Extraction form for predictors

| **INFORMATION TO EXTRACT** |  |
| --- | --- |
| First author |  |
| Year of publication |  |
| Study type | 🗌 clinical trials 🗌 cohort case-control  🗌 prospective 🗌 retrospective |
| Context | 🗌 Pain Multidisciplinary Clinic, Describe:  🗌 Other, Describe |
| Follow-up (in months) |  |
| Duration |  |
| Evaluation points |  |
| Participants’ selection |  |
| Inclusion period |  |
| Sample size |  |
| Lost to follow-up [n (%)] |  |
| Participants’ characteristics |  |
| Age (in years) [Mean (SD)] |  |
| Male gender [n (%)] |  |
| Pain chronicity definition |  |
| CHARMS and TRIPOD risk bias scores |  |
| Multivariable analysis | 🗌 Yes; Used method  🗌 No |
| Outcomes measured | 🗌 Pain intensity, describe  🗌 Disability, describe  🗌 Return to work, describe  🗌 Psychological wellbeing, describe  🗌 Quality of life, describe |
| Variables assessed |  |
| Association measures used | 🗌 p-value  🗌 Odds Ratio  🗌 Risk Ratio  🗌 Hazard Ratio  🗌 Other, Describe |
| Results in univariable analysis |  |
| Results in multivariable analysis |  |

CHARMS: CHecklist for critical Appraisal and data extraction for systematic Reviews of prediction Modelling Studies; TRIPOD: Transparent reporting of a multivariable prediction model for individual prognosis or diagnosis: The TRIPOD statement; SD: Standard Deviation

Table S3 Extraction form for multivariable models

| **INFORMATION TO EXTRACT** |  |
| --- | --- |
| First author |  |
| Year of publication |  |
| Study type | 🗌 clinical trials 🗌 cohort case-control  🗌 prospective 🗌 retrospective |
| Context | 🗌 Pain Multidisciplinary Clinic, Describe:  🗌 Other, Describe |
| Follow-up (in months) |  |
| Duration |  |
| Evaluation points |  |
| Participants’ selection |  |
| Inclusion period |  |
| Sample size |  |
| Lost to follow-up [n (%)] |  |
| Participants’ characteristics |  |
| Age (in years) [Mean (SD)] |  |
| Male gender [n (%)] |  |
| Pain chronicity definition |  |
| CHARMS and TRIPOD risk bias scores |  |
| Multivariable analysis | 🗌 Yes; Used method  🗌 No |
| Outcomes measured | 🗌 Pain intensity, describe  🗌 Disability, describe  🗌 Return to work, describe  🗌 Psychological wellbeing, describe  🗌 Quality of life, describe |
| Statistical methods used |  |
| Variables assessed |  |
| Variables included in the model |  |
| Accuracy measures used | 🗌 c-statistic  🗌 R^2^  🗌 AUC  🗌 Other, Describe |

Figure S1 Future PRISMA flow chart for the systematic review process


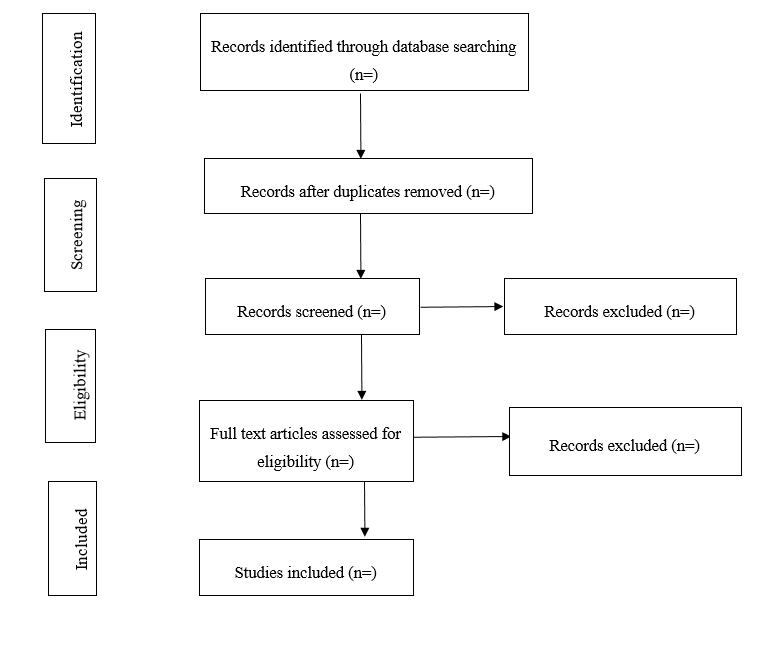

Supplement: Supplementary file 1 — Table S1. Search terms used in the different search engines. Table S2. Extraction form for predictors. Table S3. Extraction form for multivariable models. Figure S1. Future PRISMA flow chart for the systematic review process. (DOCX 46 kb) [file 13643_2018_818_MOESM1_ESM.docx]
